# Supplementary material for: The Intracellular Symbiont Wolbachia pipientis Enhances Recombination in a Dose-Dependent Manner
Source: Insects. 2020 May 6;11(5):284. doi: 10.3390/insects11050284 (PMC7290356; doi:10.3390/insects11050284)
Supplement: Supplementary file 1 [file insects-11-00284-s001.docx]

**Figure S1.** – Schematic of backcrossing to introduce *w*MelPop into the DGRP-320 background.

**Figure S2A.** – *Wolbachia* titer quantification for infection in background DGRP-320 when infected with strain wMelPop compared to strain wMel. Quantification based on qPCR across eight samples targeting the *wsp* locus of *Wolbachia.* Fold increase in *Wolbachia* quantification similar to what has been published for strain *w*MelPop*.* See methods for more detail.


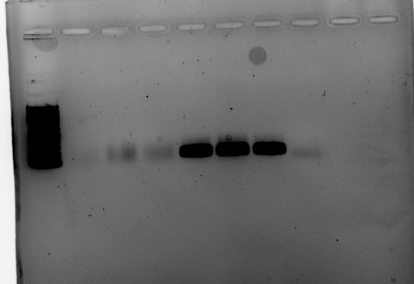


**Figure S2B. –** Verification of *Spiroplasma* infection by PCR. Lanes 2-4 are the uninfected OreR-modENCODE stock, lanes 5-7 are the *Spiroplasma*-infected OreR-modENCODE flies, lane 8 is the water-only negative control. Lane 1, 100bp ladder.

**Table S1.** List of stocks, their genotypes, and infection status for flies used in this study.

| **BDSC Stock number or this study** | **Background/Genotype** | **Infection Status** |
| --- | --- | --- |
| BDSC 1509 | *y^1^v^1^* | *Uninfected* |
| BDSC 433 | *vg^1^bw^1^* | *Uninfected* |
| BDSC 496 | *e^4^wo^1^ro^1^* | *--* |
| BDSC 29654 | DGRP-320 | *w*Mel |
| BDSC 28134 | DGRP-83 | *Uninfected* |
| BDSC 65284 | *w^1118^* *y^+^Y, sc^8^Y, Dp(1;Y)sc^8^* | *w*MelPop |
| BDSC 5 | Oregon-R-C | *w*Mel |
| BDSC 25211 | Oregon-R-modENCODE | *Uninfected* |
| This study | Oregon-R-C | *Uninfected* |
| This study | Oregon-R-modENCODE | *w*Mel |
| This study | Oregon-R-modENCODE | *Spiroplasma poulsonii MSRO* |
| This study | DGRP-320 | *Uninfected* |
| BDSC 29654 | DGRP-320 | *w*MelPop |
